# Phytocompound Mediated Blockage of Quorum Sensing Cascade in ESKAPE Pathogens

**DOI:** 10.3390/antibiotics11010061

**Published:** 2022-01-05

**Authors:** Sreejita Ghosh, Dibyajit Lahiri, Moupriya Nag, Ankita Dey, Soumya Pandit, Tanmay Sarkar, Siddhartha Pati, Zulhisyam Abdul Kari, Ahmad Razali Ishak, Hisham Atan Edinur, Rina Rani Ray

**Affiliations:** 1 Department of Biotechnology, Maulana Abul Kalam Azad University of Technology, Haringhata 741249, West Bengal, India; sreejita.ghosh@gmail.com; 2 Department of Biotechnology, University of Engineering and Management, Kolkata 700156, West Bengal, India; dibyajit.lahiri@uem.edu.in (D.L.); moupriya.nag@uem.edu.in (M.N.); 3 Department of Pathology, Belle Vue Clinic, Kolkata 700017, West Bengal, India; ankita.dey16061996@gmail.com; 4 Department of Life Sciences, Sharda University, Noida 201310, Uttar Pradesh, India; sounip@gmail.com; 5 Department of Food Processing Technology, Malda Polytechnic, West Bengal State Council of Technical Education, Government of West Bengal, Malda 732102, West Bengal, India; tanmays468@gmail.com; 6 NatNov Bioscience Private Limited, Balasore 756001, Odisha, India; patisiddhartha@gmail.com; 7 Skills Innovation and Academic Network (SIAN) Institute-ABC, Balasore 756001, Odisha, India; 8 Faculty of Agro Based Industry, Universiti Malaysia Kelantan, Jeli 17600, Kelantan, Malaysia; zulhisyam.a@umk.edu.my; 9 Center of Environmental Health and Safety, Faculty of Health Sciences, Universiti Teknologi MARA, Puncak Alam 42300, Selangor, Malaysia; 10 School of Health Sciences, Health Campus, Universiti Sains Malaysia, Kubang Kerian 16150, Kelantan, Malaysia

**Keywords:** ESKAPE pathogens, quorum sensing, QS inhibitors, bacterial pathogenesis

## Abstract

Increased resistance of *Enterococcus faecium*, *Staphylococcus aureus*, *Klebsiella pneumoniae*, *Acinetobacter baumannii*, *Pseudomonas aeruginosa*, and *Enterobacter* sp. (ESKAPE) pathogens against various drugs has enhanced the urge for the development of alternate therapeutics. Quorum sensing (QS) is a density dependent cell-to-cell communication mechanism responsible for controlling pathogenicity with the regulation of gene expression. Thus, QS is considered a potential target for the development of newer anti-biofilm agents that do not depend on the utilization of antibiotics. Compounds with anti-QS effects are known as QS inhibitors (QSIs), and they can inhibit the QS mechanism that forms the major form in the development of bacterial pathogenesis. A diverse array of natural compounds provides a plethora of anti-QS effects. Over recent years, these natural compounds have gained importance as new strategies for combating the ESKAPE pathogens and inhibiting the genes involved in QS. Different pharmacognostical and pharmacological studies have been carried out so far for identification of novel drugs or for the discovery of their unique structures that may help in developing more effective anti-biofilm therapies. The main objective of this review is to discuss the various natural compounds, so far identified and their employed mechanisms in hindering the genes responsible for QS leading to bacterial pathogenesis.

## 1. Introduction

Over the years various, selective pressures on pathogenic bacteria have resulted in the employment of different ways to adapt various types of environmental nooks. One such adaptive approach is formation of biofilm, which is a consortia of bacterial cells that remain embedded within a self-secreted extracellular polymeric substance (EPS) helping in the process of surface attachment [1,2,3]. QS plays a pivotal role in the development of biofilm [4]. The sessile microcolonies that are associated with the biofilm structure portray an elevated level of adaptive resistance to antibiotics or antibacterial drugs in comparison to planktonic counterparts [5]. Adaptive resistance against antibiotics acts as a global threat in the treatment of biofilm-associated acute and chronic infections such as surgical wound infections, nosocomial pneumonia, infections in wounds due to burns, uro-catheter infections and pneumonia caused due to ventilation [6]. Thus, formation of biofilms is actually responsible for causing various problems in food industries, health-care sectors and other medical fields [7,8]. Amongst them, the ESKAPE (*Enterococcus faecium*, *Staphylococcus aureus*, *Klebsiella pneumoniae*, *Acinetobacter baumannii*, *Pseudomonas aeruginosa*, and *Enterobacter* sp.) bacteria are accountable for a majority of the nosocomial diseases and are capable of evasion of the biocidal action of antimicrobial agents. For this very reason, the overuse and misuse of antibacterial drugs led the ESKAPE bacteria to easily ‘escape’ the actions of these drugs and cause different chronic diseases, which are not easily cured. So, alternative ways to treat and cure these diseases caused by ESKAPE pathogens are of great importance to safeguard human health. Such looming health risk has restimulated the surge for the examination of new sources of anti-biofilm agents with utmost priority [9]; and therefore, several natural resources are explored, which include different phytocompounds and microbial metabolites. Until now, many natural compounds have been isolated and identified possessing antibacterial attributes [10], of which a number of plant compounds are identified.

Phytocompounds are preferred for their relatively non-toxic nature, biocompatibility, easy availability, biodegradability, and eco-friendliness [11]. Different plant parts of *Teucrium polium* [12], *Thymus musilii* Velen [13], *Carum copticum* [14], *Salvadora persica* [15], and Syzygium jambos [16] are found to have antibacterial activities. Pteridophytes such as *Tectaria coadunata* [17], different species of the genus *Selaginella* [18], and *Adiantum philippense* [19] are noteworthy for their therapeutic potential. Some members of gymnosperms, namely *Pinus strobus,* and *Pinus pinastar* are known for their antimicrobial phytocompounds [20]. Moreover, many members of the plant family *Lamiaceae* are found to have medicinal properties [21].

Although many of the abovementioned herbal sources with antimicrobial activities do not show anti-biofilm efficacies, precisely targeting QS signals, bioactive compounds obtained from plants such as *Taraxacum*, *Tussilago* and *Scutellera* have been reported to show an anti-QS effect against biofilm formation in ESKAPE microbes [22]. These QS inhibiting phytocompounds either disrupt the pathways or inhibit the genes responsible for QS in biofilm forming bacteria including the ESKAPE. To find out some suitable alternative therapeutic measures, a thorough insight is required to discuss the anti-biofilm activities of the phytocompounds, their strategies to disrupt quorum sensing ability to address the nosocomial disease-causing deadly organisms, which may be effectively used to combat and cure chronic biofilm-related infections and there lies the rationale of the present review, whereby we intend to focus on the mechanisms by which various natural compounds can inhibit QS, thereby regulating biofilm formation and their effectiveness against ESKAPE bacteria. Such newly identified natural compounds are considered as promising therapeutic agents to combat and cure chronic biofilm-related infections.

Plant derived inhibitors of quorum sensing have been reported by many workers. High-specificity phytochemicals directly interact with QS activators through orchestrating gene expression programs. Pharmacognostical and pharmacological studies on various natural products have been performed to discover new therapeutic measures to prevent infection among drug-resistant bacterial pathogens over the period 1981–2002. However, a surge of publication on bioactive phytochemicals with quorum quenching activities was observed to have initiated from 2015 onwards. Medicinal plants, offering an attractive repertoire of phytochemicals with biofilm eradicating potential, have recently received considerable attention as a new source of safe and effective QS inhibitory substances. Few books are also published on allied topics. A moderate number of articles are being published by Science Citation Indexed Academic Journals with high impact, the majority of which are research articles dealing with exploration of new herbal sources with quorum sensing blocking activities. However, most of these works have targeted a particular microbe (bacteria or yeast). Some reviews have dealt with the chemical background of the anti-biofilm phytocompounds, while others have described their action on one or two bacteria. Searching relevant keywords clearly reflects the fact that review reports on phytocompounds with quorum sensing blocking activities reached a peak in recent years around 2018 (about 13 in 2021 till date; 10 in 2020; 5–6 per year in 2017–2019). Although a number of papers are available on phytocompounds with anti-biofilm efficacy, bibliometric analysis indicates the necessity of a systematic analysis of their respective effectivity against multidrug resistant pathogenic bacteria such as ESKAPE pathogen.

## 2. ESKAPE Pathogens: A Global Menace

Antimicrobial resistance (AMR) is a global threat to public health. This AMR is acquired by bacteria through horizontal gene transfer (HGT) via chromosomes, genetic mutations, transposons, plasmids, and some other mobile genetic elements [5,23]. The first-rate class of opportunistic pathogens that are considered as a universal threat to mankind are known as ESKAPE, that includes *Enterococcus faecium*, *Staphylococcus aureus*, *Klebsiella pneumonia*, *Acinetobacter baumannii*, *Pseudomonas aeruginosa*, and *Enterobacter* sp. Thus, it can be said that AMR can be found in both Gram-negative and Gram-positive bacterial strains. The rate at which AMR is being developed in these pathogens is alarmingly increasing. *A. baumannii*, *K. pneumonia*, and *P. aeruginosa* are resistant to carbapenem, and members of *Enterobacteriaceae* and *S. aureus* are partially resistant to vancomycin and fully resistant to methicillin and thus they are called methicillin resistant *S. aureus* (MRSA). All these resistant bacterial classes are also known as superbugs. Such pathogens are known to cause lethal diseases among immunocompromised and severely ill individuals because of improper treatment [24]. Hence, the effects of ESKAPE are devastating.

### 2.1. Enterococcus faecium

*E. faecium* is a spherical (coccus) Gram-positive bacterium occurring in chains/pairs and is generally responsible for causing nosocomial septicemia amongst immunocompromised patients. βlactam antibiotics such as penicillin and different last resource antibiotics are ineffective against *E. faecium*. Resistance to vancomycin, has in particular led to the enhanced development of *Enterococci* strains with vancomycin resistance. A gene in vancomycin resistant *Enterococci* (VRE) codes for a putative Enterococcal surface protein helping to form more thick biofilms [25]. This leads to the development of infections such as bacteremia, urinary tract infections (UTI), endocarditis, and intra-abdominal infections. Enterococcal bacteria are now considered the third most frequent nosocomial pathogen causing 14% of hospital-acquired contaminations in the United States (US) from the year 2011 to 2014, and apart from nosocomial infections, are also known to cause community-acquired endocarditis [25].

### 2.2. Staphylococcus aureus

*S. aureus* is a spherical (coccal) Gram-positive bacterium generally found in the skin microbiota of humans and is mostly harmful amongst immunocompromised individuals. Since it is normally present within the skin microflora of humans, it can cause infections only by penetrating cuts, wounds, or bruises, which are regions not inhabited by it. *S. aureus* is responsible for causing medical implant infections by formation of biofilms, which are not easily cleared by treatment with antibiotics. Around 25% of strains of *S. aureus* can secrete the exotoxin TSST-1 causing toxic shock syndrome. MRSA are the largest group of *S. aureus* to have developed resistance to β-lactam antibiotics. Resistance against β-lactam antibiotics are acquired by the expression of *mecA* encoding a protein with low affinity for binding to penicillin [26]. Thus, they are known to cause many healthcare associated infections such as infections in prosthetic devices and in infective endocarditis. Pathogenic and β-lactam resistant strains are a few of the major causative agents of soft tissue and skin infections. Many strains have also gained resistance to vancomycin, due to its overuse in treating *S. aureus* infections [27].

### 2.3. Klebsiella pneumoniae

*K. pneumoniae* is a rod-shaped bacillus Gram-negative bacterium. One third amount of all infections caused by gram-negative bacteria is due to this microbe. They are responsible in causing septicemia, UTI, cystitis, infections in surgical wounds, endocarditis, and pneumonia. It also causes pyogenic liver abscess, necrotizing pneumonia and endogenous endophthalmitis. Infections caused due to *K. pneumoniae* leads to higher mortality rates, prolonged period of hospitalization and treatments of exorbitant rates. Strains that are carbapenem resistant *K. pneumoniae* (CRKP) are known to develop β-lactamases making them resistant towards usually used antibiotics such as carbapenem [28].

### 2.4. Acinetobacter baumannii

*A. baumannii* is a purely aerobic, Gram-negative, non-fastidious, non-fermenting, oxidase-negative, catalase-positive coccobacillus (pleomorphic) bacterium known to cause almost 2% of all nosocomial infections. 45% of the strains of this lethal opportunistic pathogen have been found to be multidrug resistant (MDR) [29]. The most caused infections by it are pneumonia associated with ventilation and central-line associated infections in bloodstream. Because of their ability of biofilm formation, desiccation resistance and existence of major pathogenic properties such as glycoconjugates, surface adhesions and their system of secretion help *A. baumannii* to survive under extreme unfavorable conditions [29].

### 2.5. Pseudomonas aeruginosa

*P. aeruginosa* is a Gram-negative gamma-proteobacterium having a very slightly permeable outer membrane and multi-transport systems providing it an innate resistance to several antibiotics. It can employ diverse mechanisms such as efflux pumps, porin channel alteration, β-lactamases and modifications of the target for developing resistance towards antimicrobial substances. Patients suffering from cystic fibrosis (CF) are at a higher risk of developing this infection due to its capacity of biofilm formation and persistent cells inside the lungs [30]. *P. aeruginosa* is immensely resistant to fluoroquinolones due to the point mutations on topoisomerase IV or DNA gyrase.

### 2.6. Enterobacter sp.

*Enterobacter* sp. are facultatively anaerobic, rod-shaped, Gram-negative bacteria and belong to the family of *Enterobacteriaceae*. Patients who are immunocompromised or implanted with implantable medical device (IMD), or on support of mechanical ventilation, are mostly prone to acquire respiratory or urinary tract infections caused by this pathogen. *Enterobacter cloacae* is the commonly responsible pathogen for causing 4–5% of nosocomial pneumonia, bacteremia and UTIs [31]. They are resistant towards a broad spectrum of antibiotics such as carbapenems via extended spectrum β-lactamases (ESBLs) encoded by plasmids.

## 3. Mechanism of QS

**Figure 1:**
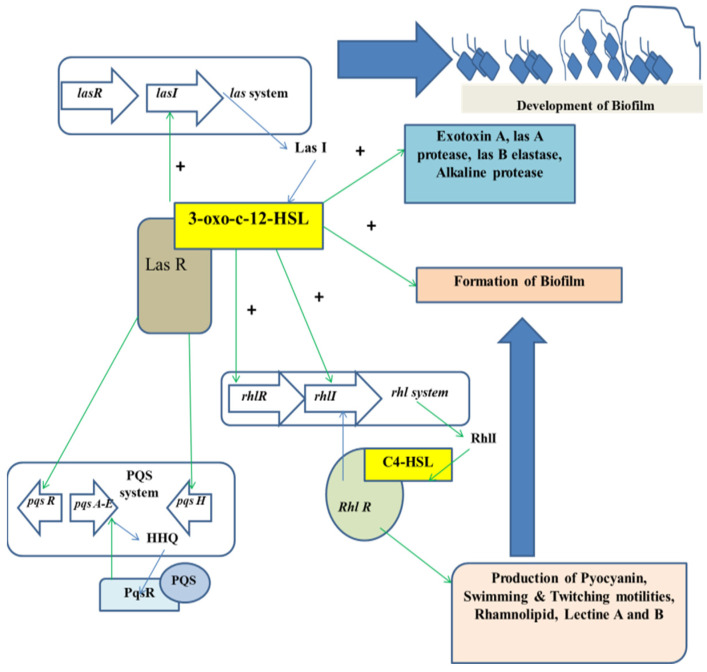
Regulation of QS in the development of biofilm.

The mechanism of QS relies on the biosynthesis, secretion, and assimilation of autoinducers (AIs) in the circumambient medium, the concentration of which is dependent on the population density of the bacterial cells. Extracellular signaling molecules and AIs accumulated within the circumambient medium in proportion to the cellular density and thus they are used for intercellular communication [32]. Their role involves the regulation of gene expression of the cells present in the community, thereby regulating a variety of responses of bacterial cells. Different physiological processes of bacteria such as motility, pathogenicity, formation of biofilms, luminescence, genetic competence development, sporulation, generation and secretion of proteolytic enzymes, biosynthesis of peptide antibiotics, and fluorescence are controlled by QS. Researchers have found that the production of signal molecules is dependent on the mechanism of autoinducing and this type of mechanism is different in Gram-positive and Gram-negative bacteria [33]. Such signaling molecules, along with their receptors, have been classified into three major groups: (i) autoinducer (AI)-2 in Gram-negative as well as in Gram-positive bacteria for interspecies communication, (ii) autoinducing peptides or oligopeptides comprising 5–34 residues of amino acids that usually take part in intercellular communication among gram-positive bacteria; a majority of these peptides are effluxed by specific systems and are modified post-translationally in different ways so that they are sensed by other cells via membrane-embedded receptors, which are part of 2-component regulatory system and (iii) *N*-acyl homoserine lactones (AHLs) that differ in length and state of oxidation of the acyl side chain and are secreted by Gram-negative bacteria in order to monitor their density of population in QS-mediated gene expression control and the signals produced by the members of the LuxI protein family (Figure 1).

### 3.1. QS Mediated by AI-2

This system of QS-mediated biofilm formation is found in both Gram-positive and Gram-negative bacteria. AI-2 is also known as furanosyl borate diester. This molecule has the capacity to switch between intra-and inter-species signalling on the basis of density and concentration at threshold [34]. AI-2 is chemically called a furanosylborate diester that is produced by the members of LuxS protein family [35]. In Gram-positive bacteria, the precursor peptide molecules of AI are altered and exported out of the cell via ATP-binding cassette (ABC) exporter complex. As the peptide AI concentration reaches the threshold value, the protein in the sensor kinase will be activated and carry out phosphorylation of the protein regulating the response, and this phosphorylated protein will bind to the target promoter leading to gene regulation by QS. Contrary to this, in Gram-negative bacteria, AIs are secreted and freely diffused out of the cell. Once the AI concentration reaches the threshold value, a positive loop of feedback is generated, causing more biosynthesis of AIs. As the AI concentration enhances in proportion to the increase in bacterial population, then after reaching a specific point, these molecules again diffuse in the bacteria for regulating specific gene transcription triggering the production and secretion of pathogenic factors, antibiotics, and form biofilms.

### 3.2. QS Mediated by Secretion of Peptide

The molecules responsible for QS in Gram-positive bacteria are based on the biosynthesis of oligopeptide from the peptide. These molecules are known as auto-inducing oligopeptides (AIPs), which are utilized as signaling molecules in QS sensing. Such AIPs are produced into the extracellular environment and they possess an affinity of binding to the histidine (His) membrane receptor kinase [36]. The AIP molecular expression is dependent on the gene family of agrD. After the AIPs are expressed, the conformation of the agrB protein bound to the membrane is altered by the incorporation of thiolactone and so they may be effluxed to the circumambient media in the form of oligopeptide. As the extracellular AIP concentration attains threshold, these molecules bind to the agrC receptor (membrane bound receptor kinase). The transmembrane transduction of signals produces the auto-phosphorylated AgrA, thereby triggering various signaling pathways bringing about the expression of agrBDCA protein. This system activation is based on the concentration of cell density. As the density of the cell increases, the system of agr will activate, thereby switching the physiological states of bacteria on the proteases and toxin production causing adherence, commensalism, aggression, and invasion [37].

### 3.3. QS Mediated by AHLs

In case of Gram-negative bacteria such as *P. aeruginosa*, RhlR-I and LasR-I systems take part in the QS regulation. The AHL molecule biosynthesis is carried out by a certain enzyme known as lactone synthase or LuxI. This enzyme is secreted within the extracellular medium. Once the cell density is increased or attained quorum, the AHL concentration in the extracellular medium reaches a critical concentration in this stage and the AHLs diffuse into the cells, thereby interacting with the transcriptional regulators [38]. In *P. aeruginosa*, AHL molecules are produced in two different forms, which are C4-HSL and 3OC12-HSL. Each form of AHL molecule binds to their respective transcriptional regulators, that is RhlR to C4-HSL, and LasR to 3OC12-HSL, respectively. The complexes thus formed activate several transcriptional regulators such as toxA, LasB, and LasI. The family gene regulator activation is also dependent on the concentration of the AHL molecules.

Alteration of physiological processes is controlled through AHLs inducing the expression of the QS genes [39]. Thus, all the AHLs found so far consist of an acyl chain having an even number of atoms of carbon ranging from 4–14 in terms of length and attached to the homoserine lactone moiety [40]. The constituents of the AHL regulated QS system belong to the LuxI and LuxR protein families. LuxI is responsible for the generation of AHLs, whereas LuxR is known to repress or activate certain gene transcription such as those of the virulent genes.

### 3.4. QS Mediated by Other Systems

Sometimes, a natural modification in the system of QS may be observed. There exist some strains of bacteria that do not synthesize self-induced molecules or peptides, while they respond with respect to the auto-induced signals produced by other bacteria. For example, *E. coli* contains a signaling molecule known as SdiA (a molecule which is homologous to LuxR) in response to the corresponding signals produced by other bacteria. Apart from this, *Burkholderia cepacia* responds to the QS signals of *P. aeruginosa* through CF signal molecules [41].

## 4. Formation of Biofilm and Its Relationship with Microbial Pathogenicity

### 4.1. Biofilm Formation in Association with QS

Formation of biofilms is one of the major reasons for the development of MDR strains of bacteria. Most of the microbial infections among humans and animals are due to the biofilm forming abilities of microbes and these infections cannot be easily treated with antimicrobial substances. Each life cycle of a biofilm consists of four main stages: primary attachment of bacterial cells, formation of bacterial colonies, growth of bacterial cells and synthesis of ECM, and finally the biofilm maturates releasing the bacterial cells to find other new niches for their growth. The substratum surface is just like the polymeric matrix found in hosts composed of proteins, exopolysaccharides, nucleic acids, and different substances allowing the bacterial cells to attach. Proteins such as SasG and Aap, present on the cell surface, take part in the initial attachment of the cells of *S. epidermidis*, and the G5 domain, present the Aap protein, helps in intercellular attachment [42]. In addition to this, extracellular constituents such as extracellular protein for glucan binding, proteins that are exposed on the surfaces and the glycosyltransferases, such as GtfH, GtfG and GtfE are responsible for cell adhesion. A transpeptidase called sortase A (Srt A) helps in anchoring proteins on the cell’s surfaces, eliciting extracellular colonization and forms biofilms in infections caused by *S. aureus*, a Gram-positive bacteria [43]. Hence, inhibitors acting against such proteins involved in adhesion need to be increasingly developed so that they can raise a good capability of antimicrobial and anti-biofilm effects. The attached bacteria will then differentiate to form micro-colonies. After the biofilm matures, there develops a complicated matrix architecture with water channels to allow nutrient influx and waste efflux [44].

ECM constitutes proteins, DNA, and carbohydrates. For example, TasA (a fibrous protein), TapA, and exopolysaccharides are significant components that take part in biofilm formation in *Bacillus subtilis* and the active expression of these matrix constituents require the presence of spermidine [45]. Various growth conditions such as pH value and availability of oxygen influence gene expression and functions. Diminished concentration of oxygen inside the biofilm may cause programmed cell lysis (PCL) and lead to the formation of biofilms in *S. aureus* [46]. This advancement is because of SaeRS and SrrAB mediated upregulation of AtlA murein hydrolase finally releasing the cytosolic DNA. Additionally some studies were performed on genome-wide scrutiny of the genes involved in biofilm formation for biosynthesis of purine and ClpYQ protease [47]. Once the biofilm matures, the bacterial cells are dispersed out of the biofilm and are free to develop new attachments, forming a new biofilm network.

The intercellular communication system of QS has led to the development of biofilms in Gram-negative as well as in Gram-positive bacterial species. The mechanism playing behind biofilm formation has been intensely studied. QS allows the bacterial cells to sense the density of population by recognizing and measuring the concentration of specific self-generated signal molecules produced by the microbial community [48]. In the meantime, gene expression in bacteria is altered and co-operative responses are activated via activation signaling pathways when the density of the population is sufficiently high for inducing the accumulated signal level in the environment. These genes code a repository of pathogenic factors such as proteases, exoenzymes, pyocyanin and elastases.

### 4.2. Signal Molecule-Associated Biofilm Formation

Gram-positive bacteria produce AIs within the environment. On increasing AI concentration, AIs start binding to the kinase receptors present on the bacterial membrane for signal transmission to the correlated transcription elements eventually activating the expression of related genes such as RNAIII and accessory gene regulator (Agr). The Agr system is considered the most fundamental QS system in Gram-positive bacteria. The Agr process in the most common Gram-positive bacteria, *S. aureus* has been widely studied, resulting in important findings such as pathogenic factor production including the production of toxins (alpha-toxin, phenol-soluble modulins or PSMs and delta toxins or hld) and degradative exoenzymes such as proteases Spl, SspB and SspA [49].

Contrary to this, AI called AHL is generally secreted by Gram-negative bacterial communication. These AHLs bind to the cytoplasmic receptors for modulating the expression of targeted genes when AHL concentration in the bacterial community is elevated. The traditional system of QS in Gram-negative bacteria consists of the presence of LuxI/LuxR transcription factors, which are activated by AHLs to produce pathogenic factors such as lectin, pyocyanin, proteases, elastase, and toxins. Different Gram-negative bacteria produce different Ais such as CAI-1, *Pseudomonas* quinolone signal (PQS), and AI-2 and various associated QS/gene receptors such as RhlI/RhlR, LasI/LasR, LuxPQ and CqsS [50].

Thus, it can be said that QS is responsible for forming the biofilm network for providing an in-born defense against external factors such as antibiotics and host immune system. Intensive investigations on the intercellular communication process and the complex biofilm architecture helps to develop new targets and strategies for the identification of new therapeutics and QSIs against biofilm-mediated infections.

## 5. Phytocompounds with Anti-QS Effects

**Figure 2:**
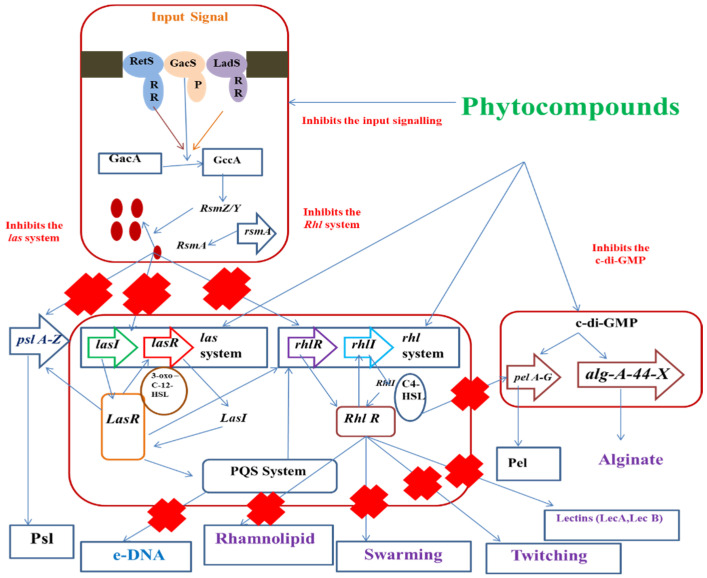
Phytocompound mediated inhibition of QS cascade.

Many phytocompounds have anti-biofilm activities in vitro. The anti-biofilm activities of natural compounds are mostly based on certain aspects such as inhibition of cell attachment and cell adhesion, suppression of polymer formation, reduction in the production of pathogenic factors and interruption of ECM biosynthesis (Table 1, Figure 2).

### 5.1. Phytochemicals

**Figure 3:**
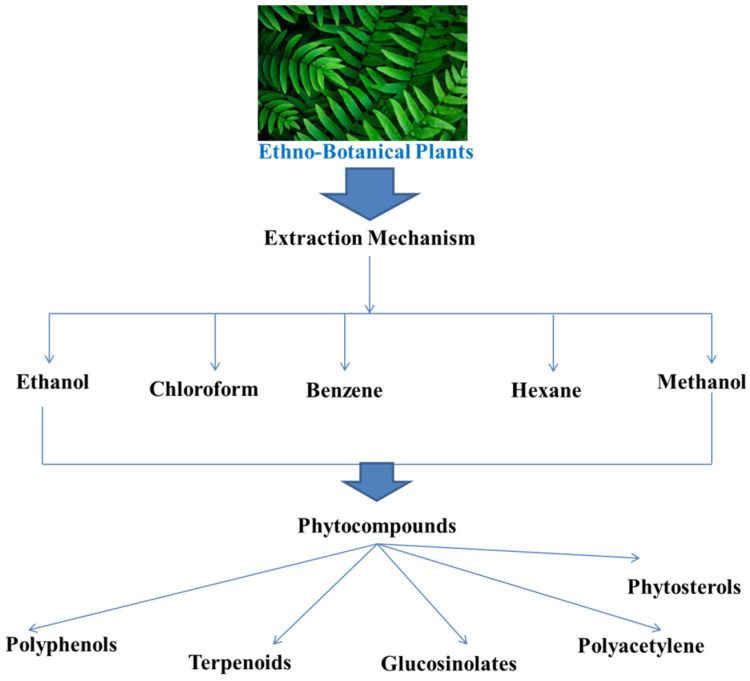
Plant derived compounds as effective anti-biofilm agent.

Natural compounds derived from plants are mainly secondary metabolites, which are either derived by oxygen substitution or phenols. These secondary metabolites have many beneficial effects, including antimicrobial characteristics. Major classes of compounds possessing antimicrobial activities derived from plants are phenolic acids, phenolics, saponins, quinones, tannins, flavonoids (FLs), terpenoids, coumarins, and alkaloids [51,52]. Due to differences in their chemical composition and structure, these compounds have varied anti-biofilm activities. The plants are a source of a group of compounds such as polyphenols, terpenoids, phytosterols and many more that have proved to have potential quorum quenching activities (Figure 3). Moreover, compounds such as oridin, naringenin, ursolic acid, salicylic acid, methyl eugenol, cinnamaldehyde and edible fruit extract are also known to show anti-biofilm activities (Figure 3).

#### 5.1.1. Alkaloids

Anti-biofilm effects of piperine and embelin were investigated on the biofilm synthesis property of *Streptococcus mutans* by means of microtiter plate assay [53]. It was seen that piperine, at a concentration of 0.0407 ± 0.03 mg/L, showed greater biofilm inhibition efficacy in comparison to that of embelin at a concentration of 0.0620 ± 0.03 mg/L. These substances inhibit the signal molecules, and their receptors present in the QS system that causes formation of biofilms.

Hordenine was first ever alkaloid reported to have the capacity of effective reduction of pathogenic factor release and QS-associated gene expression in *P. aeruginosa*, strain PAO1 [54]. The anti-biofilm effect of hordenine is mainly because it can serve as a competitive inhibitor of the QS-dependent signaling molecules and can even ward off food-borne pathogens. Gold nanoparticles (AuNPs) can be conjugated with hordenine to form hordenine-AuNPs, which displays strong anti-biofilm activity against *P. aeruginosa* strain PAO1, demonstrating that NPs, when combined with anti-biofilm agents, enhance the anti-QS efficacies in natural compounds [55].

#### 5.1.2. Flavonoids (FLs) and Flavones

FLs are a major class of plant metabolites derived from phenylpropanoids that are divided based on their degree of oxidation of C-ring and their structural variations mainly occur due to carbon skeleton substitution through glycosylation, hydroxylation, prenylation, acylation, and methylation. Some FLs can suppress the nucleic acid biosynthesis, activities of gyrase, and functions of cytoplasmic membrane, type IV topoisomerase and metabolism of energy [56]. FLs mediate the intercellular communication system between the host leguminous plants with their respective rhizobia.

Baicalein, which is a flavone at micromolar concentrations, inhibited QS-dependent biofilm formation in *P. aeruginosa* strain PAO1 and also brought about proteolytic degradation of *Agrobacterium tumefaciens* signal receptor for QS in TraR of *E. coli* [57]. Scholars have screened many FLs from citrus plants for their abilities to interrupt the QS-mediated biofilm synthesis and bioluminescence process [58]. The results obtained revealed that a compound called naringenin reduced bioluminescence induction and QS signals AI-2 and HAI-1 thereby inhibiting biofilm formation in *E. coli* 0157: H7. Above all, the 3 type III secretion system of genes involved in intercellular communication is controlled and down-regulated by naringenin.

Naringenin, flavonones, taxifolin and eriodictyol isolated from *Combretum albiflorum* extract remarkably decreased pyocyanin and elastase production in *P. aeruginosa* without inhibiting bacterial growth [59]. Taxifolin and naringenin can also reduce QS-dependent gene expression of LasR, LasI, RhlR, RhlI, LasB, LasA, RhlA, and phzA1 in *P. aeruginosa* strain PAO1. The activity of naringenin is the outcome of a combined decrease in AHL production (supported by down-regulation of *Rhl1* and *LasI* gene expression) and in the capacity of LuxR transcriptional factors to bind with the cognate receptors subsequently reducing the QS-related gene expression [59]. It is evident that the *RhlI* and *LasI* mutant strains are deficient in AHL biosynthesis because of their impaired capacities of QS gene production such as RhlA (codes for the primary protein involved in rhamnolipid production), LasB (encodes LasB elastase) and renders the phz operon (pyocyanin production) ineffective.

Catechin from *C. albiflorum* negatively influenced elastase and pyocyanin production on the formation of biofilm. It also regulated the expression of the genes involved in QS such as RhlA, LasB, LasI, LasR, RhlI, and RhlR. It may also interrupt the sensing mechanism of the QS signal molecule N-butanoyl-l-homoserine lactone (produced in response to RhlR), therefore, reducing QS factor production [60].

Researchers have observed that the naturally found cyanidin and anthocyanin notably suppressed QS-mediated phenotypes-like production of violacein (73.96%), formation of EPS (72.43%) and production of EPS (68.65%) in a concentration-dependent way in the opportunistic pathogen *K. pnuemoniae* [61]. Rosmarinic acid, isolated from sweet basil, acts as a competitive inhibitor of the bacterial ligand *N*-butanoyl-homoserine lactone (C4-HSL) and binds to the QS-regulator RhlR in *P. aeruginosa*. Further, it led to the stimulation of a significant increase in RhlR-associated transcription in vitro, in comparison to that of the C4-HSL transcription. Total anthocyanin content in *Syzgium cumini* serves as a specific inhibitor of EPS production and biofilm formation in *K. pneumoniae* up to 64.29% and 79.94%, respectively [62,63]. QS-suppression activity by *S. cumini* is due to the presence of malvidin, which decreased production of violacein and EPS and inhibited biofilm formation in *K. pneumoniae* in a concentration-dependent way.

#### 5.1.3. Limonoids and Terpenoids

Naturally found furocoumarins, extracted from grapes, displayed strong suppression of AI-2 and AI-1 effects and hindered the formation of biofilms in *P. aeruginosa* and *E. coli* [64]. Moreover, it was also found that a grape limonoid called obacunone exhibited a tough antagonistic effect against AI-2 and AHL systems, the formation of biofilm and enterohemorrhagic *E. coli* (EHEC) virulence.

Citrus limonoids are a distinctive group of major secondary metabolites (triterpenoids). Ichangin, isolimonic acid, and citrus limonoids are potential suppressors of Caco-2 cell adhesion and formation of biofilms (type III secretion mechanism) because of EHEC. They suppress flhDC and genes encoded by enterocyte effacement. Furthermore, isolimonic acid interrupted the epinephrine/AI-3-mediated activation of the intercellular-signaling pathway in QseA and QseBC [65]. Thus, isolimonic acid, in combination with ichangin and deacetylnomilinic acid glucoside, showed a strong inhibition of AI-associated intercellular signaling, subsequently reducing biofilm formation. Furthermore, isolimonic acid, in association with ichangin, increased the response regulator gene *luxO* expression.

Diterpene phytol reduced twitching and motility of flagella and biofilm formation in *P. aeruginosa*, strain PAO1. It elicited a strong inhibitory effect against the production of pyocyanin in *P. aeruginosa* [66]. An important antimicrobial agent called carvacrol isolated from oregano oil stopped the formation of biofilm in *S. aureus*, strain 0074. Moreover, it was also found to reduce civil (gene encoding the *N*-acyl-l-homoserine lactone synthase) production of violacein and activity chitinase regulated by QS [67].

#### 5.1.4. Quercetin

Quercetin is a plant-derived polyphenol that is found in many grains, vegetables and fruits [68]. It can remarkably reduce the production of pathogenic factors, including elastase, protease and pyocyanin, subsequently suppressing biofilm formation with a greater efficiency in comparison to the other plant secondary metabolites.

An 80µg/mL concentration of quercetin significantly reduces QS-mediated phenotypes such as the formation of biofilms, production of violacein, motility, production of exopolysaccharide (EPS), and production of alginate in a concentration-dependent way [62]. It serves as a competitive inhibitor for the signaling molecule in the lasR receptor path. Moreover, even at a low concentration, quercetin can remarkably suppress the formation of biofilms and pathogenic factor production, such as the production of protease, pyocyanin and elastase particularly in *P. aeruginosa*. Furthermore, levels of expression of LasR, LasI, RhlR, and RhlI were notably decreased by 68%, 34%, 50%, and 57%, respectively, in the presence of 16µg/mL quercetin. The anti-biofilm effects of quercetin in formation of biofilms and biofilm-mediated infections in *Enterococcus faecalis* exhibited a significant potential to be used as an antimicrobial substance even at a very low concentration [69]. Moreover, quercetin in conjugation with microparticles and NPs, can elicit more effective anti-biofilm effects than only using quercetin [70].

#### 5.1.5. Furanones

Halogenated furanones from marine benthic microalgae *Delisea pulchra* was the first identified anti-biofilm compound. They suppressed QS-mediated behavior by competitive binding to the proteins belonging to the LuxR family. Hence, they bring about proteolytic degradation inhibiting biofilm formation but do not kill the bacteria [71].

#### 5.1.6. Phenolic Substances

Phenolic substances are molecular compounds produced by plants. They are secondary metabolites of plants that provide protection to the plants against environmental threats. They are also called Phyto micronutrients and provide the hues and colors in fruits and leaves. Phenolic substances are one of the most important classes of phytochemicals in plants. There are over 8000 different phenols existing in plants. The common characteristic of all these phenolic compounds is the existence of at least one aromatic ring having six carbons in their chemical structures. They can be further divided into 10 various groups, depending on their fundamental chemical structures and consist of the simplest molecules such as phenolic acids to the highly complex tannins. These help in the inhibition or blocking of the underlying mechanisms of pathogenesis in various ESKAPE pathogens [72].

#### 5.1.7. Phenolic Acid

Phenolic acids are organic substances containing one phenolic hydroxyl and at least one carboxylic group. In plants, these phenolic acids are found in the form of aliphatic alcoholic esters such as glycosides, rosmarinic acid, and quininic acid. The anti-QS effects of these acid phenols are because of the presence of the number and sites of the hydroxylic groups; that is, the greater the hydroxylation, the greater the anti-QS effect. The mechanism behind the anti-QS effects of these phenolic acids may also be due to the inhibition of enzymes by oxidized substances or via chemical reactions with sulfhidric groups or non-specific reactions with the proteins involved in QS of the ESKAPE bacteria [73].

The main mechanisms by which these phytochemicals can inhibit biofilm formation and development in the target pathogens are listed in the table below (Table 1).

**Table 1:** Phytochemicals and their mechanism of clearing ESKAPE pathogenic infections.

Name of the Compound	Source of the Compound	Mechanism of Action	Organism on Which It Acts	Reference
Allicin	*Alium sativum*	Interferes with the mechanism of QS thus helps in downregulating the virulence factors.	*Pseudomonas aeruginosa*	[74]
Ajocene	*Alium sativum*	Helps in downregulating the mechanism of rhamnolipid production. It also brings about inhibition of small RNA molecules such as *rsmZ*, *rsmY*, and *rnaIII* that thereby act at the later phases of the process of QS.	*Staphylococcus aureus* and *Pseudomonas aeruginosa*	[75]
Carvacrol	*Origanum vulgare*	It brings about post-translational inhibition of *lasI*, which thereby inhibits the production of AHL	*Pseudomonas aeruginosa*	[76]
		Post-translational inhibition against lasI, which affects AHL production. It mainly acts on QS machinery.		
Emodin	*Rheum palmatum, Polygonum cuspidatum*	Helps in the downregulation of *argA*, *cidA*, *dltB*, *icaA*, *sarA*, and *sortaseA* biofilm forming genes.	*Staphylococcus aureus*	[77]
Aloe-emodin	*Rheum officinale*	Helps in downregulating the production of adhesins and polysaccharide formation.	*Staphylococcus aureus*	[78]
Hardenine	*Hordeum vulgare*	Helps in decreasing the production of AHL and also decreases the production of various types of virulence factors such as pyocyanin, protease, rhamnolipid, proviridine, and alginate. It also helps in the downregulation of *rhlI*, *lasR*, *lasI*, and *rhlR* genes.	*Pseudomonas aeruginosa*	[54]
Pulverulentone A	*Callistemon citrinus*	Prevents the formation of biofilm by inhibiting the production of staphyloxanthin.	Methicillin-resistant *Staphylococcus aureus*	[79]
(R)-Bgugaine	*Arisarum vulgare*	It inhibits the production of pyocyanin, rhamnolipid, and LasA. It also helps in inhibiting the flagellar motility.	*Pseudomonas aeruginosa*	[80]
Phytol	*Piper betle*	Helps in downregulating the QS genes, inhibits the swarming motility as well as hydrophobicity.	*Serratia mercescens*	[81]
Vitexin	*Vitex* sp	Helps in downregulating the mechanism of QS by inhibiting various genes and factors that are associated with the process.	*Pseudomonas aeruginosa*	[82]
5-Hydroxymethylfurfuryl	*Musa acuminata*	Helps in downregulating the genes associated with the process of QS and reduces the virulence.	*Pseudomonas aeruginosa*	[83]
Zingerone	*Zingiber officinale*	Helps in the mechanism of inhibiting the production of pyocyanin, elastase, and protease. It also brings about inhibition of swarming and twitching motility.	*Pseudomonas aeruginosa*	[83]
Baicalin	*Scutellaria beicalensls*	Helps in bringing about downregulation of various QS genes that include *rhlR, rhlI, lasI, lasR, pqsA, pqsR.* It also brings about inhibition in the production of protease and elastase.	*Pseudomonas aeruginosa*	[84]
Curcumin	*Curcuma longa*	Helps in downregulation of the QS genes.	*Acinetobacter baumanni*	[85]
Epigallocatechin-3-gallate	*Camellia sinesis*	Inhibits the production of various QS molecules.	*E. coli*	[86]
7-Epiclusianone	*Rheedia brasiliensis*	Helps in disrupting the biofilm and downregulates the mechanism of QS.	*Streptococcus mutans*	[87]
Hyperforin	*Hypericum perforatum*	It shows quorum quenching activity.	*Staphylococcus aureus*	[88]
Catechin	*Azadirachta indica*	Inhibits the production of pyocyanin.	*Alcaligenes faecalis*	[89]
Eugenol and linalool	*Ocimum tenuiflorum*	Inhibits the production of pyocyanin, elastase and protease.	*Pseudomonas aeruginosa*	[90]

### 5.2. Plant Extracts and By-Products

#### 5.2.1. Cuachalalate and Nutmeg Plant Extracts

Anacardic acid mixture (AAM), obtained from *Amphipterygium adstringens* and its hexane extracts, possessed anti-QS activity on the production of pathogenic substances such as pyocyanin, elastase, and rhamnolipids in the pathogen *P. aeruginosa* [91]. At concentrations of 166 and 55 µg/mL, the compounds showed 94% and 91.6% efficacy in reducing the production of violacein, respectively, without killing the bacterial cells. Additionally, AAM at concentrations of 500 µg/mL and 200 µg/mL reduced the production of elastase by 75%, rhamnolipid by 91%, and pyocyanin by 86%, respectively, in *P. aeruginosa* without reducing its viability.

Malabaricone C extracted from *Myristica cinnamonaea* bark inhibits the production of vioalcein and pyocyanin, thereby suppressing biofilm formation in *P. aeruginosa* strain PAO1 [92].

#### 5.2.2. Garlic Extract

Garlic is an opulent source of many antimicrobials. Researchers have observed that extracts from garlic made *P. aeruginosa* susceptible to destruction by phagocytosis via polymorphonuclear neutrophils (PMNs), respiratory burst and antibiotic tobramycin in a pulmonary infection mouse model [93]. Furthermore, it was also found that garlic decreased the amplification of the pathogenic factors reducing QS signal production in a UTI mouse model [94]. Garlic extracts were very effective in clearing infections caused by almost all the ESKAPE bacteria. Biological screening and rational design of all the compounds found from garlic extract revealed one of the most potent QS inhibitor compounds *N*-(heptylsulfanylacetyl)-l-homoserine lactone. This component acts as a competitive inhibitor of transcription regulators LasR and LuxR hence interfering with the QS signaling pathway. The trisulphides and disulphide metabolite obtained from garlic can bring about LuxR-associated QS inhibition in *P. aeruginosa*.

#### 5.2.3. *Cocculus trilobus* Extracts

Ethyl acetate extracts from the medicinal plants *Coptis chinensis* and *C. trilobus* stopped the adhesion of bacteria to those surfaces that are coated with fibronectin. The extracts exhibited an anti-adherence activity at the adhesion step of biofilm synthesis by inhibiting sortase (membrane enzyme) activity, and this enzyme is actually responsible for forming covalent bonds of surface proteins to that of the peptidoglycan layer in Gram-positive bacteria. Both water and ethyl acetate extracts of these two plants were investigated and it was found that the ethyl acetate portion of *C. trilobus* showed the most elevated activity in the inhibition of bacterial adhesion, via targeting the membrane enzyme sortase.

#### 5.2.4. Polyphenols from Cranberry

The fruit cranberry is considered a rich source of polyphenols. A study showed that a cranberry portion, which could not be dialyzed, was rich in polyphenols with high molecular weight [95]. These polyphenols significantly inhibited biofilm formation, thereby preventing the adhesion and colonization of the pathogenic bacteria on to the host tissues. This is because the polyphenol content of cranberry produces some enzymes that cause the disruption of the ECM, affect the proteins binding with glucan, the hydrophobicity of bacteria, reduced production of carbohydrates, coaggregation and proteolytic functions responsible for formation of biofilms. Thus, it was concluded from this investigation that cranberry polyphenols can serve as effective anti-biofilm agents and can be used to treat various oral infections such as periodontitis and dental cavities.

#### 5.2.5. Extracts from *Herba patriniae*

A lux-CDABE system of reporting was constructed for detecting six key biofilm forming genes in *P. aeruginosa* [96]. Then 36 different medicinal plant extracts were applied for screening of their inhibitory characteristics against these genes via this reported system. The results obtained showed that the extracts from *H. patriniae* exhibited remarkable inhibitory activities on a majority of the six biofilm forming genes and reduced formation of biofilms by disrupting the mature biofilm structure in *P. aeruginosa.* Furthermore, extracts from *H. patriniae* reduced production of EPS in *P. aeruginosa*. From these results, it can be concluded that *H. patriniae* extracts can be used as a novel strategy to combat infections caused by *P. aeruginosa* biofilm.

#### 5.2.6. Extracts from *Ginkgo biloba*

Extracts from *G. biloba* at a concentration of 100µg/mL remarkably inhibited biofilm formation in *E. coli*, strain O157:H7 on nylon, polystyrene and glass surfaces without affecting the growth of the bacteria [97]. It was further investigated that the main inhibition process of the *G. biloba* acids is due to the prophage and curli gene repression in *E. coli*, strain O157:H7, thereby reducing biofilm formation and fimbriae production.

In another investigation, cinnamaldehyde was found to inhibit swimming motility and formation and structure of biofilms in E. coli [98]. Cinnamaldehyde and derivatives of cinnamaldehyde disrupted the formation of biofilms, inhibited pathogenicity and stress response. It can also be said that the process of QS suppression lies in the fact that cinnamaldehyde inhibited the AI-2 associated QS by reducing DNA-binding capability of LuxR.

#### 5.2.7. Apple Extracts

One of the main components of apple extract is phloretin, which is a rich antioxidant. It was found that phloretin could effectively inhibit formation of biofilm and production of fimbriae in *E. coli*, strain O157:H7 without inhibiting planktonic cell growth [99]. Phloretin was also found to suppress attachment of *E. coli*, O157:H7 cells to the colon epithelial cells of humans and reduced the alpha-mediated inflammatory response due to tumor necrosis factor (TNF). Phloretin targets AI-2 genes (lsrACDBF), toxin genes (stx (2) and hlyE), prophage genes and curli genes (csgB and csgA) in the biofilm cells of *E. coli*, O157:H7. This survey indicated that phloretin not only acts as an anti-biofilm compound but can also act as an anti-inflammatory agent. Apart from this, phloretin displayed anti-biofilm activities against *S. aureus*, strains SA1199B and RN4220 even at a low concentration with inhibition efficacy of up to 70% by targeting the efflux proteins.

The main mechanism underlying the potential anti-QS activities of these plant by-products are enlisted below in Table 2.

### 5.3. Essential Oils (EOs)

EOs comprise of two classes of single substances, which are phenylpropanoids and terpenoids (di-terpene, sesquiterpene and monoterpenes). EOs contain compounds such as pinene, p-cymene, limonene, terpinene, sabinene, menthol, geraniol, citronellol, linalool, thymol, carvone, geranyl acetate, carvacrol, geranial, eugenynl acteate, 1,8-cineole, and neral in different chemical compositions. Additionally, phenylpropanoids consist of various aromatic substances, such as cinnamaldehyde, cinnamyl alcohol, methyl cinnamate, and eugenol.

Not all components of EOs possess anti-biofilm activities. EO (carvacrol) from *Origanum vulgare* inhibit QS system and biofilm formation in pathogenic bacteria mainly *S. aureus* (Asfour, 2018). In addition to this, other EO constituents such as limonene, linalool, gamma-terpinene, (E)-citral, eugenol and 1,8-cineole also displayed anti-biofilm activities against pathogenic bacteria.

Furthermore, EOs extracted from juniper, clary sage, marjoram and lemon are used in food processing industries for biofilm formation inhibition in *E. coli* [101].

#### Mechanism of Anti-Biofilm Activity of EOs

It is hypothesized that the possible anti-biofilm activity of EOs is due to their unique single bioactive molecules. Particularly, using single constituents for reducing the formation of biofilm is enough in the case of *E. coli* [21].

**Figure 4:**
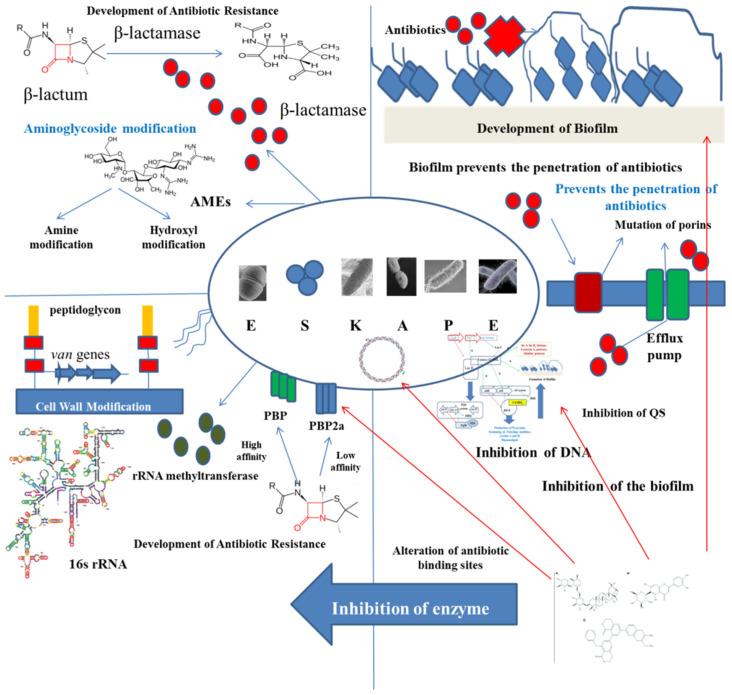
Mechanism of inhibition of QS and biofilm formation by ESKAPE pathogens with phytocompounds.

However, the synergistic effect of various components can initiate a better anti-biofilm effect of Eos, thereby reducing the resistance of several pathogenic bacteria. Some of the single components damage the microbial plasma membrane and cell walls, thereby altering their morphology and increasing permeability of the cells, but p-cymene, gamma-terpinene and carvacrol in combination can elicit a better anti-biofilm effect. This effect, due to synergism, is because of the presence of p-cymene that serves as a transport mediator for gamma-terpinene and carvacrol across the cell wall and cytoplasmic membrane in pathogens. However, the lipophilic characteristics of several single constituents bring about degradation of the microbial cell membrane and lyse the hyphal wall (Figure 4).

### 5.4. Other Products with Anti-QS Activities

Different natural components or products such as various types of leaf extracts, methanolic portion of *Zingiber officinale*, and polyphenolic constituents from *Rosa rugose* tea, demonstrated significant inhibitory activity on the development of biofilm by QS mechanism. It was found that the ethanolic extract of *Rhodomyrtus tomentosa* leaf showed an increased inhibitory activity on the formation of biofilm in *S. aureus* than the antibacterial agent vancomycin [102]. Leaf extracts of *Hymenocallis littoralis* are composed of different bioactive compounds such as methylisoeugenol and 4-methylesculentin having antibacterial characteristics against pathogenic bacteria and inhibit biofilm formation. Polyphenolic fractions of *R. rugose* tea even displayed an anti-swarming effect on the formation of biofilm in *P. aeruginosa* PAO1 and *E. coli* K-12, via targeting the QS-associated violacein factors [103].

Over recent years, natural compounds such as isovitexin, parthenolide, and erianin found in *Dendrobium chrysotoxum* indicated inhibitory activity on cellular adherence and the binding capability of QS factors and fibronectin by targeting the enzyme SrtA or through down-regulation of staphylococcal surface protein A (SpA) and block the pathogenic factors in *P. aeruginosa* and impair the formation of biofilm [104].

## 6. Conclusions and Future Perspectives

The current antibacterial alternatives formed by a wide array of natural compounds, such as phytochemicals and plant by-products or extracts, AMPS, and BSs can act as promising candidates in treating chronic and persistent biofilm infections. To fully develop and understand such approaches, we need to know more about the molecular mechanisms involved in the interactions of host-pathogen since several important pathogen-associated systems may remain unknown. Moreover, a rapid and early diagnosis of the infections and their causative agents need to be effective so that pathogenic blockers may be administered at an early infection stage [105,106]. Natural anti-biofilm compounds can be administered in cases of diseases and surgeries, where there exist chances of developing untraceable infections on dental, bone, breast implants, and eye lenses. These compounds have been found to be structurally and functionally more developed than the traditional antibiotics or antibacterial drugs. They also possess increased stability and reliability and better anti-QS activities. Several studies on medicinal plants have described the anti-biofilm potential of QS inhibiting phytocompounds, namely flavonoids, phenols, and flavonones. Some bioactive phytocompounds can also bring about an inhibition of bacterial adhesion or can repress the genes associated with the biofilm operons in ESKAPE pathogens.

Still, more studies are needed to identify more effective natural anti-biofilm compounds. An anti-adhesion approach can act as a unique strategy to treat biofilms formed by a diverse array of bacteria, since it prevents bacterial attachment and adhesion to a cellular surface. Little research has been carried out so far in this domain, so future research in eradicating biofilms by inhibition of adhesion proteins may also show a new dimension in developing anti-biofilm compounds. Expression of curli and pilli genes is effectively down-regulated by different phytocompounds. More research in this domain or combining phytocompounds having anti-adherence properties may prove to be a better way for curing biofilm-related infections. The effect of synergistic action or combined effect of such phytocompounds, in near future, may successfully replace the use of antibiotics to combat such deadly pathogens.

## Figures and Tables

**Table 2:** Plant by-products and their anti-QS mechanism against target pathogens.

Plant By-Products and Plant Extracts	Source of the Natural Products	Anti-QS Mechanism	Target Pathogen	References
AAM	*A. adstringens*	Reduced production of pathogenic substances such as pyocyanin, elastase and rhamnolipids.	*P. aeruginosa*	[91]
Malabaricone C	Bark of *M. cinnamonaea*	Inhibits production of vioalcein and pyocyanin thereby suppressing biofilm formation.	*P. aeruginosa* PAO1	[92]
*N*-(heptylsulfanylacetyl)-l-homoserine lactone	Garlic extract	Serves as a competitive inhibitor of transcription regulators LasR and LuxR, hence interfering with the QS signaling pathway.	*P. aeruginosa*	[93]
Ethyl acetate extracts	*C. trilobus* and *C. chinensis*	Blocks the adhesion of bacteria to those surfaces, which are coated with fibronectin.	*S. aureus*	[100]
Polyphenols	Cranberry	Inhibits biofilm formation thereby preventing the adhesion and colonization of the pathogenic bacteria on to the host tissues.	Gram-negative ESKAPE pathogens	[95]
lux-CDABE reporter mechanism	*H. patriniae*	Inhibited biofilm-forming genes and disrupted the biofilm structure.	*P. aeruginosa*	[96]
Cinnamaldehyde	*G. biloba*	Inhibits swimming motility and formation and structure of biofilms.	*E. coli*	[98]
Phloretin	Apple extracts	Blocks AI-2 genes (lsrACDBF), toxin genes (*stx* (2) and *hlyE*), prophage genes and curli genes (*csgB* and *csgA*) in the biofilm cells.	*E. coli* 0157: H7	[99]

## Data Availability

The data presented in this study are available in the article.
